# Supplementary material for: Serpentine alteration as source of high dissolved silicon and elevated δ30Si values to the marine Si cycle
Source: Nat Commun. 2020 Oct 12;11:5123. doi: 10.1038/s41467-020-18804-y (PMC7550359; doi:10.1038/s41467-020-18804-y)
Supplement: Supplementary file 1 — Supplementary Information [file 41467_2020_18804_MOESM1_ESM.pdf]

Supplementary Information for

Serpentine alteration as source of high dissolved silicon and elevated $\delta^{30}Si$ values to the marine Si cycle

by

Geilert et al.

Supplementary Figures

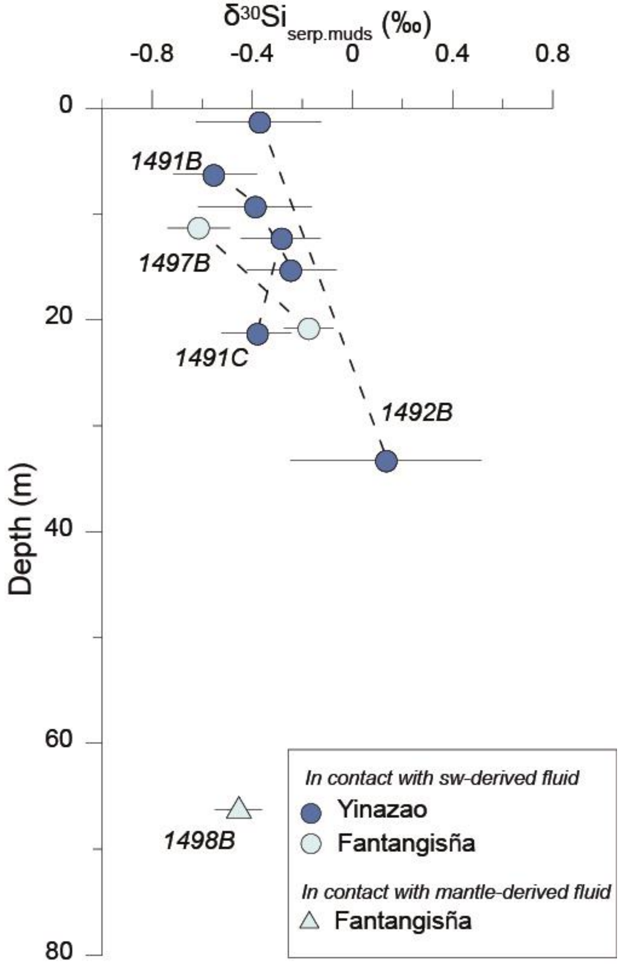

Supplementary Figure 1. The $\delta^{30}Si$ values of serpentine muds versus seamount depth (error bars are 2SD of individual measurements).

Supplementary Methods

Mass balance equations and boundary conditions: A transport-reaction model was set up to simulate the processes occurring in the surface zone of serpentinite seamounts. The 1-D pore fluid model considers molecular diffusion, upward fluid flow, bottom water intrusion in surface sediments, serpentine dissolution/precipitation and talc precipitation. The turnover of dissolved species in the pore fluid is simulated applying the following mass balance equation:

$$\Phi * \frac{\partial C}{\partial t} = \frac{\partial}{\partial x}\left(\Phi * \left(D_S * \frac{\partial C}{\partial x} + v * C\right)\right) + \Phi * \alpha * (C_{BW} - C) + \Phi * R \qquad (1)$$

with Φ: porosity, C: concentration of dissolved species in pore fluid (µmol cm^{-3}), t: time (yr), x: sediment depth (cm), D_S: molecular diffusion coefficient of dissolved species in sediment pore fluid (cm^2 yr^{-1}), v: upward fluid flow velocity of pore fluid (cm yr^{-1}), α: mixing coefficient (yr^{-1}), R: turnover rates of dissolved species (µmol cm^{-3} yr^{-1}). The model was set up for the following dissolved species: Si, ^{30}Si, B, ^{11}B, Sr, ^{87}Sr, Cl$^-$.

At the upper (x = 0) and lower boundary (x = L) of the model, constant concentrations are applied employing ambient bottom water values (C_{BW}) and concentrations in the ascending fluids (C_{FL}):

$$C\big|_{x=0} = C_{BW} \qquad\qquad C\big|_{x=L} = C_{FL} \qquad (2)$$

The model is solved using the solver for partial differential equations of MATHEMATICA (version 11.3) applying the Method-of-Lines approach. The model is integrated over time until a steady state is attained. Parameter values, depth-dependent functions and rate terms applied in the model are listed in Supplementary Tables 3- 5.

Isotope modeling: We applied and extended a previously developed isotope model to simulate the reactive transport of dissolved silica in surface sediments[1,2] using the model framework defined in Equation (1). In this model, two separate mass balance equations (Equation (1)) are set up to simulate the turnover of dissolved ^{30}Si and total Si. The isotopic composition of the pore fluid is calculated as ratio of these two compounds ($MF_{30} = {}^{30}Si/Si$). Considering the abundance of the three Si isotopes ^{28}Si, ^{29}Si and ^{30}Si, the mol fraction MF_{30} is related to the commonly used isotope ratio ($R_{30} = {}^{30}Si/{}^{28}Si$) as:

$$R_{30} = \frac{MF_{30} \cdot R_{St30} \cdot (R_{St29} \cdot (c_R - 1) - 1)}{c_R \cdot MF_{30} \cdot R_{St29} + R_{St30} \cdot (MF_{30} - 1)} \qquad (3)$$

with $R_{St30} = 0.0341465$, $R_{St29} = 0.0507446$ and $c_R = 0.51$ [1,2].

The $\delta^{30}Si$ value of the pore fluid (in ‰) is calculated from the ratio as:

$$\delta^{30}Si = \left(\frac{R_{30}}{R_{St30} - 1}\right) \cdot 1000 \qquad (4)$$

A similar approach is applied for total Sr and ^{87}Sr. Here, the mol fraction ($MF_{87} = {}^{87}Sr/Sr$) is related to the isotope ratio ($R_{87} = {}^{87}Sr/{}^{86}Sr$) as[3]:

$$R_{87} = \frac{9.43 \cdot MF_{87}}{MF_{87} - 1} \qquad (5)$$

considering the abundance of the Sr isotopes ^{84}Sr, ^{86}Sr, ^{87}Sr, and ^{88}Sr.

The boron mole fraction ($MF_{11} = {}^{11}B/B$) is related to the $\delta^{11}B$ value of the pore fluid as:

$$\delta^{11}B = \left(\frac{MF_{11}}{R_{St11}\cdot(1-MF_{11})} - 1\right)\cdot 1000 \qquad (6)$$

where R_{ST11} is the $^{11}B/^{10}B$ ratio of the NBS boric acid standard[4]. The diffusive flux of dissolved B is calculated considering the diffusion coefficients and concentration gradients of boric acid (BA, $B(OH)_3$) and borate (BO, $B(OH)_4^-$). The diffusion term for B in Equation (1) is hence expressed as:

$$D_S \cdot \frac{\partial C}{\partial x} = D_{S,BA} \cdot \frac{\partial BA}{\partial x} + D_{S,BO} \cdot \frac{\partial BO}{\partial x} \qquad (7)$$

where the concentrations of boric acid (*BA*) and borate (*BO*) are calculated from the total dissolved boron concentration (C_B) calculated in the model (Equation (1)) and the measured pore fluid pH:

$$BA = \frac{C_B}{K_B \cdot 10^{pH} + 1} \qquad BO = C_B - BA \qquad (8)$$

K_B is the dissociation constant of boric acid in seawater[5].

A similar approach is applied for ^{11}B:

$$D_S \cdot \frac{\partial C}{\partial x} = D_{S,BA} \cdot \frac{\partial\, ^{11}BA}{\partial x} + D_{S,BO} \cdot \frac{\partial\, ^{11}BO}{\partial x} \qquad (9)$$

where the concentration of ^{11}BA and ^{11}BO are calculated as:

$$^{11}BA = \frac{BO + BA \cdot K_{B11} + MF_{11} \cdot C_B \cdot \left(K_{B11} - 1\right) - SQR}{2 \cdot \left(K_{B11} - 1\right)} \quad (10)$$

$$^{11}BO = \frac{-BO - BA \cdot K_{B11} + MF_{11} \cdot C_B \cdot \left(K_{B11} - 1\right) + SQR}{2 \cdot \left(K_{B11} - 1\right)} \quad (11)$$

with:

$$SQR = \sqrt{\left(K_{B11} \cdot BA + BO - MF_{11} \cdot C_B \cdot \left(K_{B11} - 1\right)\right)^2 + 4 \cdot BO \cdot MF_{11} \cdot C_B \cdot \left(K_{B11} - 1\right)} \quad (12)$$

The isotopic equilibrium constant K_{B11} has a value of 1.0272[6].

Reaction rates: The model considers serpentine dissolution and precipitation and the precipitation of talc. Serpentine (chrysotile) equilibration with seawater was expressed applying the following stoichiometry:

$$Mg_3Si_2O_5(OH)_4 + 6\ H^+ = 3\ Mg^{2+} + 2\ Si(OH)_4 + H_2O \quad (13)$$

For talc, we use:

$$Mg_3Si_4O_{10}(OH)_2 + 6\ H^+ + 4\ H_2O = 3\ Mg^{2+} + 4\ Si(OH)_4 \quad (14)$$

The corresponding solubility constants are defined as:

$$K_{Serp} = \frac{\{Mg^{2+}\}^3 \cdot \{Si(OH)_4\}^2 \cdot \{H_2O\}}{\{H^+\}^6} \quad (15)$$

$$K_{Talc} = \frac{\{Mg^{2+}\}^3 \cdot \{Si(OH)_4\}^4}{\{H^+\}^6 \cdot \{H_2O\}^4} \quad (16)$$

where curly brackets indicate activities. Saturation states with respect to serpentine and talc are calculated as:

$$\Omega_{Serp} = \frac{(a_{Mg} \cdot [Mg^{2+}])^3 \cdot (a_{Si} \cdot [Si(OH)_4])^2 \cdot a_W}{\{H^+\}^6 \cdot K_{Serp}} \quad (17)$$

$$\Omega_{Talc} = \frac{(a_{Mg} \cdot [Mg^{2+}])^3 \cdot (a_{Si} \cdot [Si(OH)_4])^4}{\{H^+\}^6 \cdot {a_W}^4 \cdot K_{Talc}} \quad (18)$$

where square brackets indicate concentrations (in mol kg^{-1}) and a_x activity coefficients. Solubility constants and activity coefficients are calculated in PHREEQC[7] for the temperature and pressure conditions and the solution composition in the surface layer of the seamounts (4.2 °C, 400 bar, 35 PSU). Proton activities and Mg^{2+} concentrations are derived from measured data (pH values, pore fluid Mg^{2+} concentrations) while dissolved silica concentrations are dynamically calculated in the model (Equation (1)).

The rate of serpentine dissolution applied in the model is defined as:

$$R_{DSerp} = k_{Serp} \cdot (1 - \Omega_{Serp}) \qquad \text{for } \Omega_{Serp} < 1 \quad (19)$$

while precipitation rates are defined as:

$$R_{PSerp} = k_{Serp} \cdot (\Omega_{Serp} - 1) \qquad \text{for } \Omega_{Serp} > 1 \qquad (20)$$

$$R_{PTalc} = k_{Talc} \cdot (\Omega_{Talc} - 1) \qquad \text{for } \Omega_{talc} > 1 \qquad (21)$$

Kinetic constants (k_x) were constrained by fitting the model to the data.

The turnover of ^{30}Si induced by these reactions results as:

$$R_{DSerp}(^{30}Si) = MF_{30,Serp} \cdot R_{DSerp} \qquad (22)$$

$$R_{PSerp}(^{30}Si) = \beta_P \cdot MF_{30,Si(aq)} \cdot R_{PSerp} \qquad (23)$$

$$R_{PTalc}(^{30}Si) = \beta_P \cdot MF_{30,Si(aq)} \cdot R_{PTalc} \qquad (24)$$

where $MF_{30,Serp}$ is the mol fraction of ^{30}Si in serpentine and $MF_{30,Si(aq)}$ is the mol fraction of ^{30}Si in dissolved silica. The fractionation factor between the authigenic mineral formed by precipitation (β_P) and dissolved silica is defined as (Geilert et al.[1]):

$$\beta_P = \left(e^{\Delta Si/1000}\right)^{\frac{1}{1.058}} \qquad (25)$$

where ΔSi is the $\delta^{30}Si$ difference (in ‰) between the authigenic phase and dissolved silica ($\Delta Si = \delta^{30}Si_{Au} - \delta^{30}Si_{Si(aq)}$).

Boron turnover is calculated assuming that the B content of the precipitating phases is proportional to the borate concentration in pore fluid (*BO*):

$$R_{DSerp}(B) = r_{Serp} \cdot R_{DSerp} \qquad (26)$$

$$R_{PSerp}(B) = r_P \cdot BO \cdot R_{PSerp} \qquad (27)$$

$$R_{PTalc}(B) = r_P \cdot BO \cdot R_{PTalc} \qquad (28)$$

where r_{Serp} is the molar B/Si ratio in dissolving serpentine and r_P is a fitting constant defining the B content of the precipitated phase for a given dissolved borate concentration.

The turnover of ^{11}B induced by these reactions results as:

$$R_{DSerp}(^{11}B) = MF_{11,Serp} \cdot R_{DSerp}(B) \qquad (29)$$

$$R_{PSerp}(^{11}B) = MF_{11,Au} \cdot R_{PSerp}(B) \qquad (30)$$

$$R_{PTalc}(^{11}B) = MF_{11,Au} \cdot R_{PTalc}(B) \qquad (31)$$

where $MF_{11,Serp}$ is the ^{11}B mole fraction in dissolving serpentine and $MF_{11,Au}$ is the ^{11}B mole fraction in authigenic minerals (serpentine, talc) precipitating from solution. The latter mole fraction is related to the isotopic composition of dissolved borate ($M_{F11,BO}$ = ^{11}BO/BO) and is defined as:

$$MF_{11,Au} = \frac{MF_{11,BO} \cdot e^{\Delta BO/1000}}{1 + MF_{11,BO} \cdot \left(e^{\Delta BO/1000} - 1\right)} \quad (32)$$

where *ΔBO* is the δ^{11}B difference (in ‰) between boron bound in authigenic phases and dissolved borate ($\Delta BO = \delta^{11}B_{Au} - \delta^{11}BO$). With this formulation, we assume that borate rather than boric acid is taken up in authigenic phases.

Supplementary Discussion

The Cascadia Basin Si anomaly. High Si concentrations (> 150 µM Si) between 2000 and 3000 m water depths define the Northeast Pacific Silicic Acid Plume (NPSP), which originates mostly from the Cascadia Basin (about 200 µM Si)[8,9]. These high DSi concentrations are associated with high δ^{30}Si values of +1.5‰, which are the highest Si isotope signatures in the deep Pacific ocean[10–13]. Dissolution of sedimentary biogenic silica as well as hot to warm hydrothermal fluids have been excluded as fluid source on the basis of Ge/Si ratios, modeled biogenic δ^{30}Si data, benthic and hydrothermal Si fluxes, and δ^{30}Si values[14–16]. Another source of high δ^{30}Si fluids might be low-temperature alteration of serpentinites and seafloor basalts. The NE Pacific comprises several fracture zones (e.g. Mendocino Fracture Zone) and ridges (e.g. Gorda Ridge, Juan de Fuca Ridge) which can channel seawater along deep-reaching faults deep into the mantle driving serpentinization. Additionally, serpentine bodies have been observed in the forearc upper mantle at the Cascadia margin[17] and the Gorda escarpment (south of the Mendocino Fracture Zone)[18]. In an attempt to model the required δ^{30}Si flux to the basin, boundary values from Johnson et al.[8] have been taken and extended for an isotopic component (see below). The modelled δ^{30}Si value associated with a Si flux of 0.47 Tmol yr^{-1} [8] is +4.7±0.7‰. This is still higher than the average δ^{30}Si value of about +3‰ measured in Mariana seamount pore fluids (main text Fig. 7; Supplementary Table 1), however, not impossible given the highest Mariana seamount pore fluid δ^{30}Si value of +5.2‰.

Cascadia model set-up. The calculation of the Si isotope input necessary for the Cascadia Basin to sustain its high δ^{30}Si values was calculated after the box model from Johnson et al.[8] and extended for an isotopic component. The model involves a water mass inflow at Latitude 40.96 and Longitude 127.48 (F_{in}) with a Si concentration of 186 µM[8] (also calculated for published Si concentration of 192 µM[14] and 194 µM (J. Jones, pers. Comm. on 17.05.2019)). The water mass outflow at Latitude 48.59 and Longitude 127.49 (F_{out}) had a Si concentration of 196 µM[8] (also calculated for published Si concentration of 204 µM[14] and 202 µM (J. Jones, pers. Comm. on 17.05.2019)). The distance between the inflow and outflow water masses is about 848 km, the lateral range estimated from the coast to offshore with 200 km, and the water depth range 0.5 km (vertical height after Johnson et al.[8]). The volume of the box model was thus 8.5x10^{13} m^3 and the surface area for the inflow and outflow area was 1x10^8 m^2. The northward water flow velocity across the inflow and outflow area was 1.5 cm s^{-1} (473,364 m yr^{-1}) and the northward water flow across the inflow and outflow area calculated as 4.73 x 10^{13} m^3 yr^{-1} (velocity x area). The residence time of the water mass was 1.79 yr (box volume divided by the water flow through the box). The Si release to sustain the high Si concentration in the basin was derived by the difference between outflow and inflow flux, with F_{in} = 8.80 Tmol yr^{-1} (Si concentration inflow/ total basin water flow) and F_{out} = 9.27 Tmol yr^{-1} (Si concentration outflow/total basin water flow). The required Si release (Si_{re}) yielded 0.47336 Tmol yr^{-1}. The associated Si isotope value ($\delta^{30}Si_{re}$) was calculated following isotopic steady state:

$$\delta^{30}Si_{re} = \frac{(\delta^{30}Si_{out} * F_{out} - \delta^{30}Si_{in} * F_{in})}{Si_{re}} \qquad (33)$$

With $\delta^{30}Si_{out}$ = 1.57‰ and $\delta^{30}Si_{in}$ = 1.41‰ (J. Jones, pers. comm. on 17.05.2019). The calculated $\delta^{30}Si_{re}$ is thus +4.1‰. Taking the other published Si concentrations into account (see above) the calculated $\delta^{30}Si_{re}$ values resulted in +4.5‰ and +5.5‰, with an average of +4.7±0.7‰.

Supplementary Tables

Supplementary Table 1: Pore fluid pH, Si and B concentrations, $\delta^{30}Si$, $\delta^{29}Si$, and $\delta^{11}B$ isotope values as well as Sr concentrations and $^{87}Sr/^{86}Sr$ ratios.

Site	sample	depth (m)	pH	Si (µM)	$\delta^{30}Si$ (‰)	2 SD (‰)	$\delta^{29}Si$ (‰)	2 SD (‰)	B (µM)	$\delta^{11}B$ (‰)	2 SD (‰)	Sr (µM)	$^{87}Sr/^{86}Sr$	2 SD
Yinazao seamount														
U1491B	1-H-2	*3*	*7.7*	243	0.8	0.2	0.4	0.2	553	n.d.	n.d.	83.73	0.70915	1.5E-05
	2-H-2	*6*	*7.8*	283	2.7	0.1	1.3	0.2	649	38.5	0.2	86.13	0.70914	1.4E-05
	2-H-4	*9*	*7.8*	240	2.6	0.2	1.3	0.2	597	38.6	0.2	81.93	0.70914	1.5E-05
	3-H-2	*15*	*8.3*	142	5.2	0.1	2.8	0.1	631	43.5	0.2	94.59	0.70909	1.4E-05
U1491C	2-H-2	*3*	*7.7*	147	0.7	0.2	0.4	0.1	576	37.5	0.2	83.35	0.70914	1.5E-05
	2-H-4	*6*	*7.7*	80	0.4	0.1	0.3	0.1	568	38.2	0.3	84.85	0.70913	1.6E-05
	3-F-2	*12*	*7.8*	190	2.4	0.2	1.2	0.1	752	40.5	0.2	90.97	0.70907	1.5E-05
	6-F-2	*21*	*8.0*	257	3.3	0.2	1.7	0.2	758	41.6	0.3	112.1	0.70901	1.5E-05
U1492B	1-H-1	*1*	*7.7*	226	2.8	0.2	1.5	0.1	493	n.d.	n.d.	214.0	n.d.	n.d.
Fantangisña seamount														
U1498A	1-R-3	*4*	*7.8*	293	1.3	0.2	0.6	0.2	477	37.6	0.2	83.55	0.70916	4.3E-06
	5-R-2	*38*	*8.7*	bdl	n.d.	n.d.	n.d.	n.d.	*445*	42.7	0.1	*171.9*	n.d.	n.d.
	13-R-2	*114*	*7.8*	bdl	n.d.	n.d.	n.d.	n.d.	n.d.	39.8	0.1	n.d.	n.d.	n.d.
	15-R-1	*134*	*7.9*	516	1.4	0.1	0.8	0.1	396	39.3	0.3	189.4	0.70763	1.5E-05
U1498B	8-R-3	*66*	*11.2*	bdl	1.6	0.1	0.8	0.1	111	16.1	0.2	175.7	0.70539	1.4E-05
	12-R-5	*109*	*11.1*	bdl	n.d.	n.d.	n.d.	n.d.	n.d.	16.2	0.1	n.d.	n.d.	n.d.
	15-R-2	*134*	*11.2*	bdl	n.d.	n.d.	n.d.	n.d.	*84*	16.5	0.2	*193.0*	0.70505	1.5E-05

	19-R-3	*175*	*11.0*	bdl	n.d.	n.d.	n.d.	n.d.	*103*	18.0	0.2	*220.7*	0.70518	2.0E-05
U1497B	6-F-2	*21*	*8.2*	44	4.1	0.2	2.1	0.2	97	n.d.	n.d.	577.9	n.d.	n.d.

Data in italics from Fryer et al.[19]

Abbreviations: bdl: below detection limit; n.d. not detected

Supplementary Table 2: Serpentinite mud $\delta^{30}Si$ and $\delta^{29}Si$ values.

Serpentinite muds

Site	sample	depth (m)	$\delta^{30}Si$ (‰)	2 SD (‰)	$\delta^{29}Si$ (‰)	2 SD (‰)
Yinazao seamount						
U1491B	2-H-2	6	-0.6	0.2	-0.3	0.04
	2-H-4	9	-0.4	0.2	-0.2	0.1
	3-H-2	15	-0.2	0.2	-0.1	0.1
U1491C	3-F-2	12	-0.3	0.2	-0.1	0.1
	6-F-2	21	-0.4	0.1	-0.2	0.01
U1492B	1-H-1	1	-0.4	0.2	-0.2	0.2
		33	0.1	0.4	0.1	0.2
Fantangisña seamount						
U1498B	8-R-3	66	-0.5	0.1	-0.2	0.1
U1497B	6-F-2	11	-0.6	0.1	-0.3	0.1
		21	-0.1	0.1	0.0	0.1

Supplementary Table 3. Parameter values applied in the transport-reaction model

Parameter	Symbol	Value
Temperature	T	4.2 °C
Pressure	P	400 bar
Salinity	S	35 PSU
Porosity at x = 0	Φ_0	0.83
Porosity at x = infinity	Φ_f	0.30
Attenuation coefficient for porosity decrease	px	0.0012 cm^{-1}
Mixing coefficient at x = 0	α (0)	5 x 10^{-4} yr^{-1}
Depth of mixing zone	x_M	2500 cm
Intergranular upward fluid flow velocity at x = 0	v(0)	0.01 cm yr^{-1}
Molecular diffusion coefficient of dissolved Si	$D_{M,Si}$	189 cm^2 yr^{-1}
Molecular diffusion coefficient of boric acid	$D_{M,BA}$	209 cm^2 yr^{-1}
Molecular diffusion coefficient of dissolved borate	$D_{M,BO}$	183 cm^2 yr^{-1}
Molecular diffusion coefficient of Sr	$D_{M,Sr}$	139 cm^2 yr^{-1}
Molecular diffusion coefficient of Cl^-	$D_{M,Cl}$	361 cm^2 yr^{-1}
Solubility constant of serpentine	K_{Serp}	9 x 10^{34}
Solubility constant of talc	K_{Talc}	9 x 10^{23}
Activity coefficient of dissolved Si	a_{Si}	1.09
Activity coefficient of water	a_W	0.991

Activity coefficient of Mg^{2+}	a_{Mg}	0.31
Molar B/Si ratio of dissolving serpentine	r_{Serp}	5×10^{-4}
Constant defining the B content in authigenic phases	r_P	3×10^{-3} cm^3 μmol^{-1}
$\delta^{30}Si$ of dissolving serpentine	$\delta^{30}Si_{Serp}$	-0.3 ‰
$\delta^{11}B$ of dissolving serpentine	$\delta^{11}B_{Serp}$	+16 ‰
Isotopic fraction for Si in authigenic phases	ΔSi	-3.0 ‰
Isotopic fraction for borate in authigenic phases	ΔB_{borate}	-20 ‰
Kinetic constant for serpentine dissolution and precipitation	k_{Serp}	1.5 µmol cm^{-3} yr^{-1}
Kinetic constant for talc precipitation	k_{Serp}	5×10^{-4} µmol cm^{-3} yr^{-1}

Supplementary Table 4. Depth-dependent functions applied in the transport-reaction model (depth in cm)

**Parameter**	**Symbol**	**Equation**
Porosity	Φ	$\Phi = \Phi_f + (\Phi_0 - \Phi_f) \cdot \exp(-px \cdot x)$
Mixing coefficient	α	$\alpha = \frac{\alpha(0)}{1 + \exp\left(\frac{x - x_M}{200}\right)}$
Upward fluid flow velocity	v	$v = v(0) \cdot \frac{\Phi_0}{\Phi}$

Tortuosity	To	$To^2 = 1 - 2 \cdot ln(\Phi)$
Diffusion coefficient of solutes in pore fluid	D_S	$D_S = \frac{D_M}{To^2}$
Pore fluid pH	pH	$pH = \frac{7.75 - 11.1}{1 + \exp\left(\frac{x - 2500}{500}\right)} + 11.1$
Dissolved Mg^{2+} (in mmol kg^{-1})	Mg	$Mg = \frac{51.5 - 0.003}{1 + \exp\left(\frac{x - 2500}{150}\right)} + 0.003$

Supplementary Table 5: Rate terms (R) applied in mass balance equations of the transport-reaction model (in µmol cm^{-3} yr^{-1})

**Species**	**Equation**
Dissolved Si	$R_{DSerp} – R_{PSerp} - R_{PTalc}$
Dissolved B	$R_{DSerp}(B) – R_{PSerp}(B) - R_{PTalc}(B)$
Dissolved Sr	0
Dissolved Cl^-	0
^{30}Si in dissolved Si	$R_{DSerp}(^{30}Si) – R_{PSerp}(^{30}Si) - R_{PTalc}(^{30}Si)$
^{11}B in dissolved B	$R_{DSerp}(^{11}B) – R_{PSerp}(^{11}B) - R_{PTalc}(^{11}B)$
^{87}Sr in dissolved Sr	0

Supplementary References

1. Geilert, S., Grasse, P., Doering, K., Wallmann, K., Ehlert, C., Scholz, F., Frank, M., Schmidt, M., Hensen, C. Impact of ambient conditions on the Si isotope fractionation in marine pore fluids during early diagenesis. *Biogeosciences* **17**, 1745–1763 (2020).
2. Ehlert, C., Doering, K., Wallmann, K., Scholz, F., Sommer, S., Grasse, P., Geilert, S., Frank, M. Stable silicon isotope signatures of marine pore waters – Biogenic opal dissolution versus authigenic clay mineral formation. *Geochim. Cosmochim. Acta* **191**, (2016).
3. Wallmann, K. Controls on Cretaceous and Cenozoic evolution of seawater composition, atmospheric CO2 and climate. *GCA* **65**, 3005–3025 (2001).
4. Hoefs, J. *Stable Isotope Geochemistry*. (Springer-Verlag, 1997).
5. Zeebe, R. E. & Wolf-Gladrow, D. Stable Isotope Fractionation. *CO2 Seawater Equilibrium, Kinet. Isot.* 141–250 (2001).
6. Klochko, K., Kaufman, A. J., Yao, W., Byrne, R. H. & Tossell, J. A. Experimental measurement of boron isotope fractionation in seawater. *Earth Planet. Sci. Lett.* **248**, 261–270 (2006).
7. Parkhurst, D. L. & Appelo, C. A. J. *Description of Input and Examples for PHREEQC Version 3 — A Computer Program for Speciation , Batch-Reaction , One-Dimensional Transport , and Inverse Geochemical Calculations*. (U.S. Geological Survey Techniques and Methods, 2013).
8. Johnson, H. P., Hautala, S. L., Bjorklund, T. A. & Zarnetske, M. R. Quantifying the North Pacific silica plume. *Geochemistry Geophys. Geosystems* **7**, (2006).
9. Talley, L. D. & Joyce, T. M. The double silica maximum in the North Pacific. *J. Geophys. Res.* **97**, 5465–5480 (1992).
10. Beucher, C. P., Brzezinski, M. A. & Jones, J. L. Sources and biological fractionation of Silicon isotopes in the Eastern Equatorial Pacific. *Geochim. Cosmochim. Acta* **72**, 3063–3073 (2008).
11. Hendry, K. R. & Brzezinski, M. A. Using silicon isotopes to understand the role of the Southern Ocean in modern and ancient biogeochemistry and climate. *Quat. Sci. Rev.* **89**, 13–26 (2014).
12. de Souza, G. F., Slater, R. D., Dunne, J. P. & Sarmiento, J. L. Deconvolving the controls on the deep ocean's silicon stable isotope distribution. *Earth Planet. Sci. Lett.* **398**, 66–76 (2014).
13. Grasse, P., Closset, I., Jones, J. L., Geilert, S. & Brzezinski, M. A. Controls on Dissolved Silicon Isotopes along the US GEOTRACES Eastern Pacific Zonal Transect (GP16). *Global Biogeochem. Cycles* **34** (2020). doi:https://doi.org/10.1029/2020GB006538
14. Esther, T. A., Hammond, D.E., Hautala, S.L., Johnson, H.P., Schwartz, R.J., Paukert, A.N. Evaluation of the budget for silicic acid in Cascadia Basin deep water. *Deep. Res. Part I* **57**, 677–686 (2010).
15. Wheat, C. G. & McManus, J. The potential role of ridge-flank hydrothermal systems on oceanic germanium and silicon balances. *Geochim. Cosmochim. Acta* **69**, 2021–2029 (2005).
16. Gao, S., Wolf-Gladrow, D. A. & Völker, C. Simulating the modern δ^{30}Si distribution in the oceans. *Global Biogeochem. Cycles* **30**, 120–133 (2013).
17. Brocher, T. M., Parsons, T., Tréhu, A.M., Snelson, C.M., Fisher, M.A. Seismic evidence for widespread serpentinized forearc upper mantle along the Cascadia margin. *Geology* **31**, 267–270 (2003).
18. Hoover, S. M. & Tréhu, A. M. Uplift, Emergence, and Subsidence of the Gorda Escarpment Basement Ridge Offshore Cape Mendocino, CA. *Geochemistry, Geophys. Geosystems* **18**, 4503–4521 (2017).

19. Fryer, P., Wheat, C. G., Williams, T. & Expedition 366 Scientists, T. Mariana Convergent Margin and South Chamorro Seamount. *Proc. Int. Ocean Discov. Program, 366 Coll. Station. TX (International Ocean Discov. Program)* (2018).
